# Enhanced imaging of lipid rich nanoparticles embedded in methylcellulose films for transmission electron microscopy using mixtures of heavy metals

**DOI:** 10.1016/j.micron.2017.03.019

**Published:** 2017-08

**Authors:** Jalal Asadi, Sophie Ferguson, Hussain Raja, Christian Hacker, Phedra Marius, Richard Ward, Christos Pliotas, James Naismith, John Lucocq

**Affiliations:** a School of Medicine, University of St Andrews, St. Andrews, Fife, KY16 9TF, UK; b Biomedical Sciences Research Complex, North Haugh, University of St. Andrews, St. Andrews, Scotland, UK

**Keywords:** CL, Cardiolipin, DMPC, dimyristoyl-phosphatidylcholine, DHPC, dihexanoyl phosphatidylcholine, EM, electron microscopy, LRPN, lipid rich nanoparticle, MC, methyl cellulose, PE, phosphatidyl ethanolamine, PG, phosphatidyl glycerol, PTA, phosphotungstic acid, STA, sodium silicotungstate, UA, uranyl acetate, Bicelles, Phospholipids, Membranes, Lipids, Methylcellulose, Uranyl acetate, Phosphotungstic acid, Sodium silicotungstate, Negative stain, Liposomes, Nanodiscs

## Abstract

Synthetic and naturally occurring lipid-rich nanoparticles are of wide ranging importance in biomedicine. They include liposomes, bicelles, nanodiscs, exosomes and virus particles. The quantitative study of these particles requires methods for high-resolution visualization of the whole population. One powerful imaging method is cryo-EM of vitrified samples, but this is technically demanding, requires specialized equipment, provides low contrast and does not reveal all particles present in a population. Another approach is classical negative stain-EM, which is more accessible but is difficult to standardize for larger lipidic structures, which are prone to artifacts of structure collapse and contrast variability. A third method uses embedment in methylcellulose films containing uranyl acetate as a contrasting agent. Methylcellulose embedment has been widely used for contrasting and supporting cryosections but only sporadically for visualizing lipid rich vesicular structures such as endosomes and exosomes. Here we present a simple methylcellulose-based method for routine and comprehensive visualization of synthetic lipid rich nanoparticles preparations, such as liposomes, bicelles and nanodiscs. It combines a novel double-staining mixture of uranyl acetate (UA) and tungsten-based electron stains (namely phosphotungstic acid (PTA) or sodium silicotungstate (STA)) with methylcellulose embedment. While the methylcellulose supports the delicate lipid structures during drying, the addition of PTA or STA to UA provides significant enhancement in lipid structure display and contrast as compared to UA alone. This double staining method should aid routine structural evaluation and quantification of lipid rich nanoparticles structures.

## Highlights

• Uranyl acetate/tungsten double stains are proposed for imaging lipid rich nanoparticle in TEM.
• Combined with methylcellulose embedment, the technique enhances membrane contrast.
• The technique works for liposomes, nanodiscs and bicelles.
• The double staining should improve quantification of lipid rich nanoparticles.

## 1 Introduction

Lipid rich nanoparticles (LRNPs) are of considerable importance in nanomedicine and basic research (see Hernández-Rocamora et al., 2014a, Poulos et al., 2015, Landreh and Robinson, 2015). An extensive range of LRNPs can be synthesized in the laboratory including liposomes, nanodiscs (Borch and Hamann, 2009) and bicelles (Poulos et al., 2015, Harroun et al., 2005, Rubio et al., 2011a, Vestergaard et al., 2015). Liposomes have long been proposed as vehicles for drug and cosmetic delivery and have recently been standardized well enough for clinical trials to be performed (Sercombe et al., 2015). Nanodiscs and bicelles are more recent innovations and are commonly used as platforms for studies of membrane proteins (Borch and Hamann, 2009, Bayburt and Sligar, 2010, Hernández-Rocamora et al., 2014b, Schuler et al., 2016, Murakami, 2012, Ward et al., 2014), wherein direct binding of lipids to hydrophobic pockets of these systems has been shown to play a major role in their regulation (Pliotas et al., 2015, Pliotas and Naismith, 2016) and structural integrity (Tanaka et al., 2015). Naturally occurring lipid structures include enveloped viruses, multivesicular body-derived exosomes and lipoprotein particles. Exosomes are a strong focus of interest (Théry et al., 2006) because they can carry diagnostic signatures of disease into body fluids, which then act as liquid biopsies. The imaging of naturally occurring lipid structures will not be considered further here.

Currently a range of indirect biophysical techniques is used for visualizing, characterization and quantifying LRNPs (Van der Pol et al., 2013). These include NMR, differential scanning calorimetry, polarization microscopy, small angle X ray scattering (SAXS), differential light scattering (DLS) and nanoparticle tracking analysis (NTA). Electron microscopy (EM) is also a powerful technique because of its superior resolution and direct structural display. One EM method of choice is cryo-EM, where rapid freezing affords direct observation of lipid structures. However, this is still not routine and is methodologically complex (Cheng et al., 2015, Henderson, 2015). Importantly it does not yet provide the comprehensive display of particle populations needed for reliable conclusions to be drawn about the particle populations that are present prior to freezing. A more accessible method is conventional negative stain EM (Harris and De Carlo, 2014). This works by drying down heavy metal stains onto surfaces coated with the objects of interest, thereby highlighting their morphology (Harris and De Carlo, 2014). Over the past few decades, negative stain has provided considerable amounts of valuable information on molecular structures. However, visualization of large (10 nm to 500 nm) LRNPs, that is the concern of the current study, is more challenging because of distortion, collapse and denaturation of the structures, which occurs during the drying process. Even the smallest lipid structures, such as nanodiscs (9–16 nm largest diameter), produce variable imaging results (Katayama et al., 2010, Imura et al., 2014).

One approach that could offer improvements in EM imaging of LRNPs is embedment in hydrophilic films composed of methylcellulose (MC). MC was first used routinely in EM for supporting aldehyde fixed ultrathin cryosections during drying (Tokuyasu, 1978, Tokuyasu, 1980, Griffiths et al., 1984). MC films prevent collapse of sectioned membrane structures and allow quite homogeneous contrast when heavy metals are included in the films. Since the early results on cryosections, a few studies have shown the utility of MC films in supporting and contrasting small isolated membrane bound organelles such as endosomes (Lucocq et al., 2001), phagosomes (Kjeken et al., 2004) or exosomes (Van der Pol et al., 2013); demonstrating proof of principle for imaging synthetic lipid rich nanoparticles. Importantly, MC films are easy to prepare, are relatively homogeneous and allow imaging of all particles, which is a key feature needed for appropriate sampling and quantification (Hacker et al., 2016).

In this report we investigate the use of MC films in combination with novel mixtures of heavy metals for embedding and supporting a range of lipid structures. We demonstrate that MC films containing mixtures of uranyl acetate and tungsten stains (phosphotungstic acid (PTA) or sodium silicotungstate (STA)), provides striking improvements in the contrast of lipid structures that are superior to either stain applied individually. This MC embedding/staining procedure provides comprehensive visualization of adsorbed particles and restricts collapse of these soft biological structures. The MC double-stain method has the potential to substantially improve routine characterization and quantification of a wide range of synthetic as well as naturally occurring membrane structures.

## 2 Methods

### 2.1 Materials

Pioloform (polyvinyl butyral) was obtained from Agar Scientific (Essex, UK).

Octyl glucoside purchased from Melford Laboratories Ltd (Suffolk, UK). *E. coli* Polar Lipid extract was obtained from Avanti Polar Lipids (Alabaster, AL). HEPES, potassium nitrate (KNO_3_), ammonium molybdate, uranyl acetate, phosphotungstic acid, sodium silicotungstate, methylamine tungstate, ruthenium red, MC (25 centipoise) and sodium nitrate (NaNO_3_) were purchased from Sigma Aldrich (UK).

### 2.2 Preparation of nanodiscs, bicelles and liposomes

Nanodiscs and bicelles were prepared as described previously (Ward et al., 2014). Liposomes were prepared using the bio-bead-modulated detergent removal method (Marius et al., 2008) with minor modification. Briefly, 10 mg of *E.coli* polar lipid extract in chloroform was dried under a nitrogen stream and any remaining solvent was removed using a vacuum desiccator. The lipid film formed was hydrated in 1 ml of Buffer A (10 mM HEPES, 100 mM (KNO_3_), 60 mM (NaNO_3_), pH 7.4) containing 40 mM β-d-octylglucoside. This lipid suspension was sonicated to clarity for approximately 10 min. The suspension was gently stirred at room temperature for 15 min and the detergent was slowly removed by two hourly additions of 100 mg SM2 Bio-beads (Bio-rad Laboratories, Hemel Hampstead, UK). The liposomal mixture was then passed through a Sephadex G50 (coarse) column, which was pre-equilibrated with buffer A. The liposomes were eluted in 2.5 ml aliquot as a cloudy suspension.

### 2.3 Stains and support films

Ammonium molybdate, uranyl acetate, phosphotungstic acid, methylamine tungstate and ruthenium red were kept in glass-walled containers and stored in the dark. The pH value of stains was adjusted using NaOH and or HCl. Stains were centrifuged prior to use at 13,800 × *g* for 10 min to remove aggregates. The stain solutions were used at the following concentrations (w/v) and pH values respectively: ammonium molybdate 2% pH 6.3, uranyl acetate 3% pH 3 or 3.5, sodium phosphotungstate 1% pH 7.2, sodium silicotungstate pH 7.2 1%, methylamine tungstate 2% pH 7, and ruthenium red 1% pH 8.1. Standard plastic support films were made using 1% pioloform (Agar Scientific, Stansted, Essex, UK) and copper hexagonal 100 or 150-mesh grids applied to the film.

### 2.4 Methylcellulose, staining procedure and contrast assessment

In this paper components of staining mixtures are expressed as volumes of set stock concentrations expressed in % w/v. This allows for the easy adjustment of stains using whole numbers and allows comparison with the most widely used concentrations, proportions and volumes of stain components in routine EM.

A 2% w/v solution of MC was prepared by mixing MC powder with deionized water and made up to 100 ml. The solution was heated to boiling and the solution cooled to 4 °C and stirred overnight. It was then centrifuged in a fixed angle rotor (Beckman 45Ti at 100,000 × *g*, at 4 °C for at least 2 h). The supernatant was removed without disturbing the pellet and was kept refrigerated at 4 °C.

Grids were incubated on 8–10 μl of solutions containing nanodiscs, bicelles or liposomes for 2–15 min on ice to allow them to adhere. The grids were then washed in 3–4 droplets of deionized water on ice and transferred to MC stain mixtures on ice for 5 or 10 min. A tungsten wire loop made with 0.3 or 0.2 mm gauge wire was then used to lift the grid from the drop of stain. Excess staining solution was then removed using a filter paper before by air-drying at room temperature.

The standard MC contrasting technique for sections was a mixture of 100 μl 3% uranyl acetate and 900 μl parts 2% MC. In initial experiments the amounts of each stain (ammonium molybdate, UA, PTA or STA) was varied by simply adding 50, 100, 150 or 200 μl of each stain either singly, or as a double staining mixture to the standard 900 μl volume of MC. When stains were applied in sequence, the grids were first incubated on a MC stain mixture for 5 min before a wash on a single drop of water and incubation on a second MC stain mixture (all at ice temperature). Such stain sequences used UA followed by PTA or STA (or vice versa). In the standardized double staining protocol experiments using PTA and STA, 100 μl of stain 3% uranyl acetate was added to 900 μl volume of MC followed by an addition 100 μl volume containing deionized water and varying volumes of PTA and STA solutions. In experiments aimed at reducing precipitation in UA/PTA double stain, a standard total volume of 900 μl contained 700 μl MC and 200 μl of deionized water. For conventional negative staining, samples were incubated in droplets of stain and then drained from the edge and air-dried.

Stereology was used to quantify the extent to which structures were obscured by precipitates. Eight micrographs were taken at systematic random locations across the methylcellulose film on an EM grid and each images overlaid with a systematic array of points. The fraction of total points falling on precipitates was then computed.

Orientations of nanodiscs were quantified after embedding using either 50 μl UA or 150 μl PTA added to varying quantities of MC. In each image the number of stacked/tilted nanodiscs were recorded and the percentage of each reported. Nanodiscs were selected in quadrats using the forbidden line rule (Gundersen, 1977).

Images were recorded on a JEOL 1200EX microscope fitted with an Orius 200 digital camera (Gatan; Abingdon, UK) and contrast was assessed from dm4 files in imageJ (NIH, Boston MA, USA) along intercepts set orthogonally across clear membrane profiles of vesicles and covering portions of the background film. Data was presented as range, coefficient of variation and absolute grey scale values.

## 3 Results

### 3.1 Liposomes

**Fig. 1:**
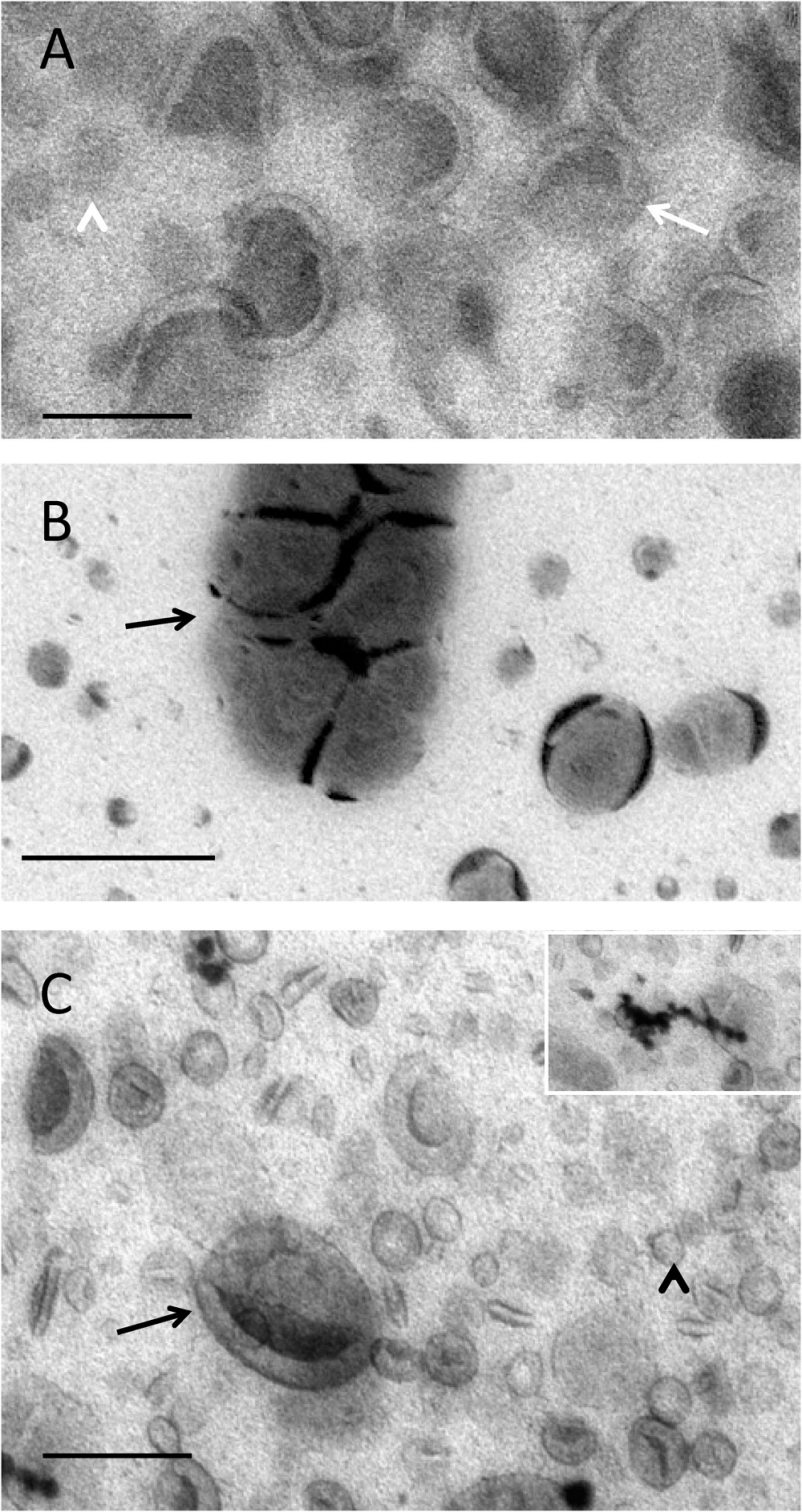
Liposomes. Liposomes in methylcellulose films containing uranyl acetate (A), phosphotungstic acid (B) and uranyl acetate/phosphotungstic acid mixture (C). Note the difference between the clarity of display of the membrane structures visualized with UA and UA/PTA (A and C respectively; larger structures, arrows and smaller structures, arrowheads). Inset in C shows characteristic precipitates generated in the mixed UA/PTA stain. The clumping of vesicles that occurs with PTA (arrow in B) alone is not apparent when the UA/PTA mixture is used. Bars 100 nm.

Liposomes were used as a model preparation for testing staining combinations and were prepared from *E. coli* polar lipid extract obtained from Avanti, containing 67% phosphatidyl ethanolamine (PE) and 23% phosphatidyl glycerol (PG) and 10% cardiolipin (CL). Conventional negative stain produced areas of intense background contrast interspersed with areas in which vesicles displayed classical comma or cup shaped morphology, typical of vesicle collapse (data not shown). The standard MC staining procedure for cryosections uses uranyl acetate and this provided a more consistent result than negative stain, revealing limiting membranes of the larger vesicles (Fig. 1A). Here the vesicle border exhibited positive contrast while the background staining in the MC film situated between the vesicles showed a comparable density to that found over the body of the vesicle themselves. Larger vesicles (approximately 500 nm in size) showed the greatest tendency for cup shaped or distorted morphology, which was interpreted as due to collapse in a direction orthogonal to the support. The smaller vesicles were often obscured due to lack of luminal contrast or membrane staining and these vesicles appeared as indistinct circular “shadows” within the film, lacking a cup shaped appearance. Varying the levels of uranyl acetate in the stain mix did not improve the contrast or membrane staining efficiency, although the best contrast between structures and the background was observed in the thinnest films.

When phosphotungstic acid was included in the MC as a single contrasting agent, more intense staining of the liposome surfaces was observed (this was likely due to ionic binding to the positively charged head groups in PE; Fig. 1B). Often, a thick layer of stain on the vesicle surface obscured the morphology of the vesicle membranes. The background staining over regions of the film situated between the vesicles was much lower than observed with UA alone. In contrast to UA staining, we observed aggregation of liposomes into large clumps, which likely reflects movement of the vesicles after attaching to the grid (see discussion). Other metal-based electron stains (ammonium molybdate, sodium phosphotungstate, methylamine tungstate or ruthenium red), when used singly, did not produce improvements in liposome display as compared to either uranyl acetate or PTA staining.

**Fig. 2:**
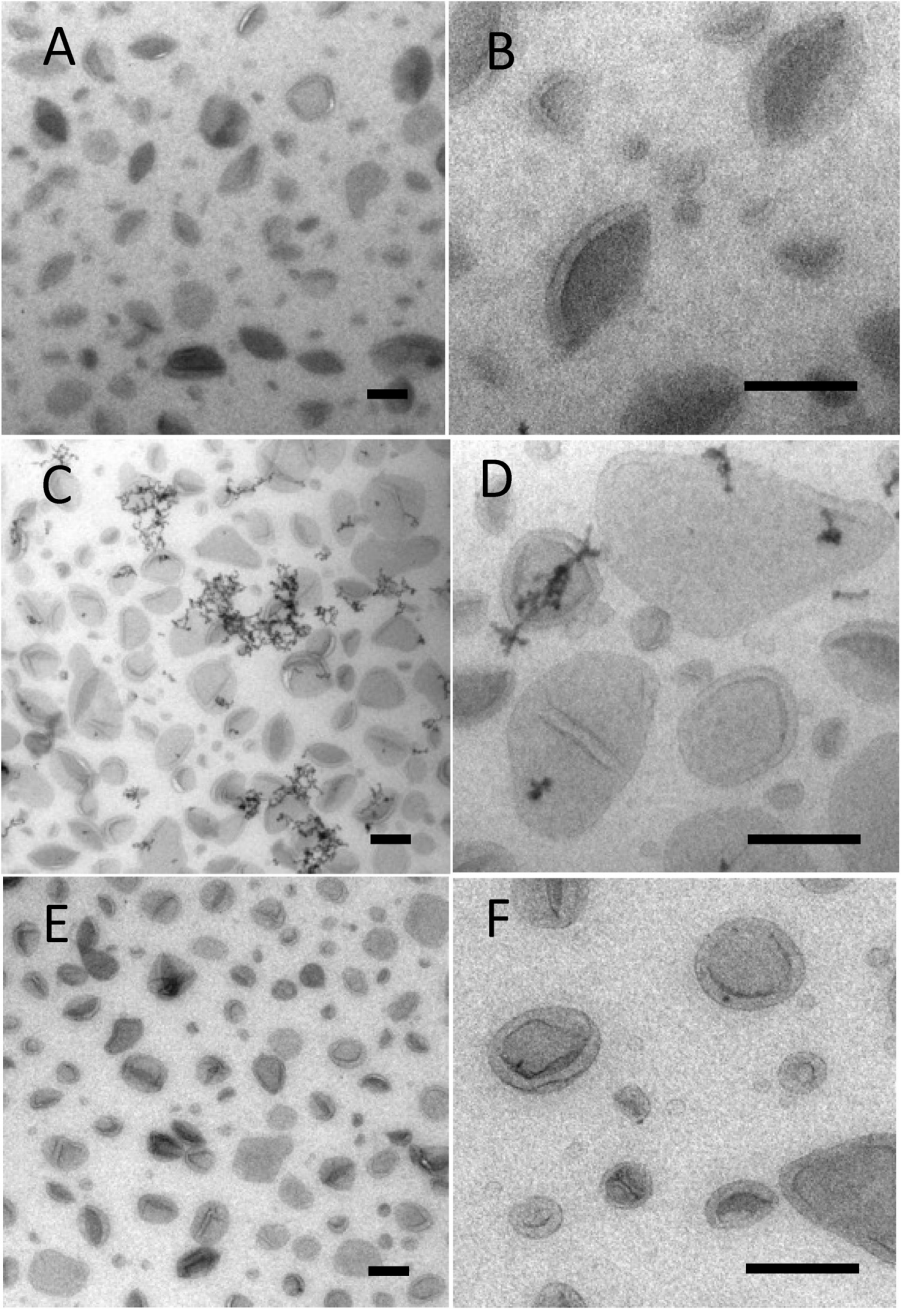
Comparison of single UA stain with mixed double stains on liposomes adsorbed to EM support films. Single UA (A and B); UA/PTA (C and D) and UA/STA (E and F). (A, C and E) are low magnification overviews and (B D and F) higher magnification. Note the precipitates observed with UA/PTA and their absence with UA/STA (E and F). Bars 100 nm.

In contrast to single staining with UA or PTA, we found that a mixture of UA and PTA in MC produced striking vesicle display (Figs. 1C; 2 A–D). The UA/PTA combination produced strong staining of the outer vesicle surfaces of both large and small liposomes − the smallest of which were now clearly displayed. Interestingly, the background electron density of the MC film situated between the vesicles was much reduced compared to single UA-staining. Further, the aggregation of vesicles seen with PTA staining alone did not appear as marked, indicating that UA inhibits this effect.

Electron dense precipitates were a feature of UA/PTA double staining (inset in Figs. 1C; 2C and D) and were present irrespective of the type of nanoparticle preparation. The precipitates appeared as linear strings or complex forms with granular or crystalline morphology and measured up to a few hundred nanometers across. The extent to which these precipitates/crystals obscured underlying details was analyzed using stereology. The fraction of liposomes that were obscured was estimated to be 3.6% (826 points counted; 150 μl each of UA and PTA).

We found that the quantity of precipitates decreased when the amount of PTA in the staining mixture was reduced. For example at 25 μl of PTA the precipitates were almost absent in the liposome preparation (900 μl MC/100 μl UA/25 μl PTA/75 μl H_2_O; not shown). In addition, precipitates could be reduced further by decreasing the volume of MC in this staining mixture to 700 μl (producing thinner MC films; data not shown). Thus, it was possible to reduce the level of precipitates by titrating PTA and/or MC downwards.

We argued that the precipitates observed with UA/PTA double staining were due to phosphorus moiety of PTA interacting with uranyl ions. We therefore tested another tungsten-containing stain, sodium silicotungstate (STA), which lacks phosphorus (Fig. 2E and F). When STA was used with UA in place of PTA, the precipitates were now almost absent, while the overall contrast was comparable to UA/PTA. The recommended starting protocol would therefore be a double stain mix with UA/STA in MC (9 parts 2% MC/1 part 3% UA/0.25 parts 1% STA/0.75 parts H_2_O). In some cases, we found that reducing thickness of wire loops from 0.3 mm to 0.2 mm further improved the clarity and contrast of images across all staining protocols with UA/STA producing the clearest images of the smallest liposome structures.

**Fig. 3:**
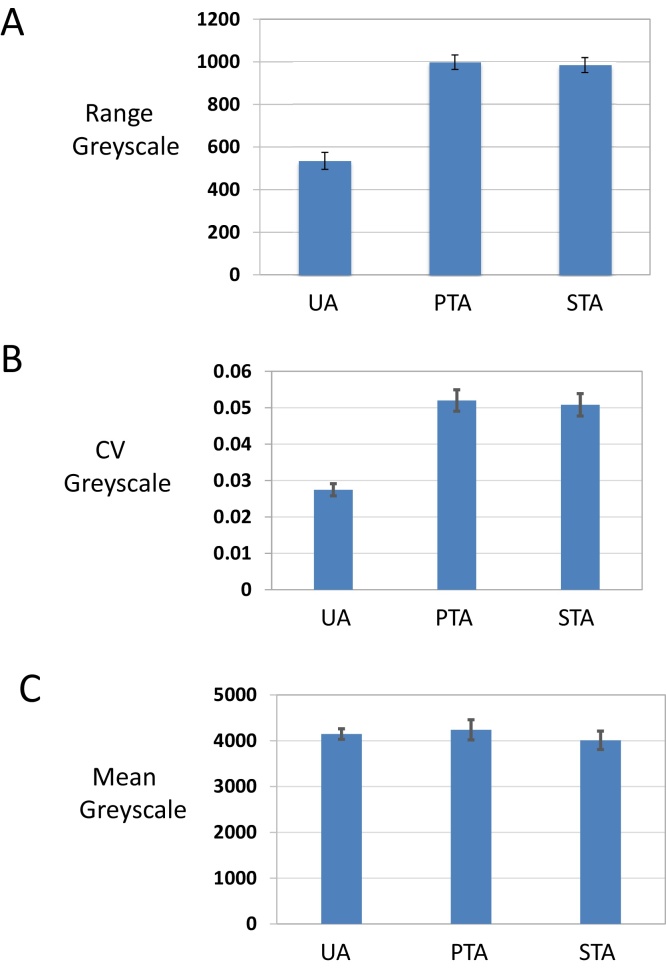
Analysis of contrast. Randomly selected particles from randomly taken micrographs were analyzed using imageJ software. Dm4 files were displayed and grey scale values measured along linear intercepts placed orthogonal to, and across, the most prominent membrane profile of the sampled vesicle. Care was taken to include stretches of surrounding film, which occupied up to one half of the total intercept length. Only precipitate-free particles were included in the analysis. (A) – range of grey scale, (B) − coefficient of variation (CV) of grey scale values and, (C) – mean of grey scale values. Error bars represent the standard error of the mean, N = 13 in each case.

To quantify the improvements in contrast we analyzed greyscale images using imageJ (Fig. 3). Plotting values across the edge of liposome vesicles, the range of grey scale values was approximately 2 fold larger for UA/PTA and UA/STA as compared to UA alone (Fig. 3A). Furthermore, the coefficient of variation for the grey scale values for the mixed stains was again two fold larger than for UA alone (Fig. 3B), while the average intensity of grey scale values was similar (Fig. 3C).

### 3.2 Bicelles

For molar ratios of dimyristoyl-phosphatidylcholine (DMPC) and dihexanoyl phosphatidylcholine (DHPC) between 2 and 5, the complex morphology of bicelles depends on temperature. Below the main chain melting temperature of 23 °C, disc-like micelles predominate. At higher temperatures, a chiral nematic phase of cigar or wormlike forms occurs (Vestergaard et al., 2015). In this study bicelles were first adsorbed to the EM support film at or below 22 °C and washed at the same temperature before they were embedded in MC stain mixtures at ice temperature. The expectation was to find a mixture of both disc-like forms and cigar-shaped structures.

**Fig. 4:**
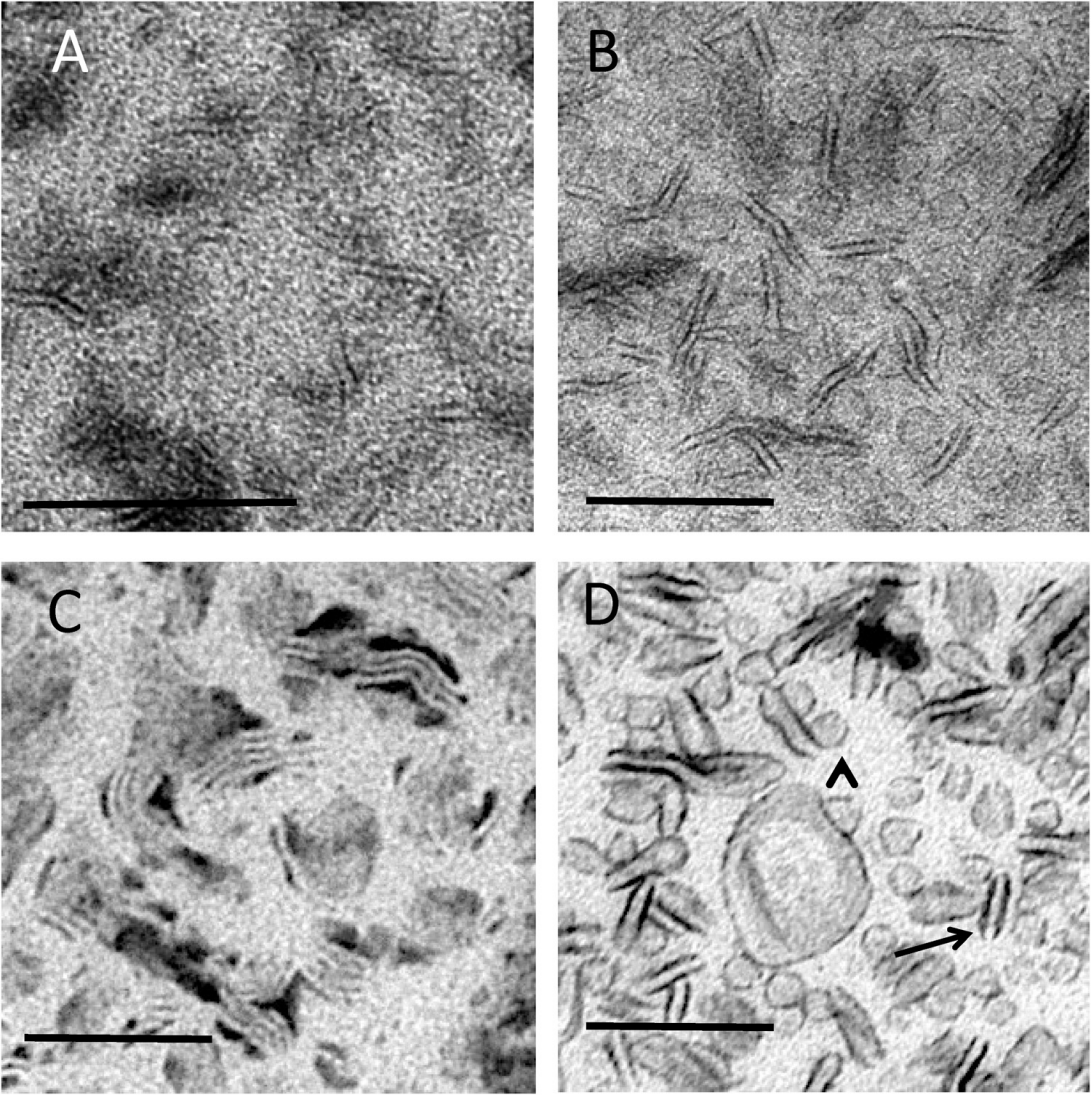
Bicelles. Bicelles were adsorbed to EM support films at 22°C and stained using (A) UA alone (classic negative stain), (B) UA in MC, (C) PTA in MC and (D) UA/PTA mixture in MC. The smaller structures are difficult to discern in (A) and (B) and undergo clumping in (C). Smaller round profiles (arrowhead) and the edges of the wormlike extended structures (arrows) are best displayed in the mixed UA/PTA stains, (D). Bars, 100 nm.

Conventional negative staining of bicelles showed evidence for wormlike or cigar shaped forms with some indistinct circular forms present in the background (Fig. 4A). When the same preparation was embedded in MC containing UA, the wormlike structures were more obvious and appeared to be interspersed with indistinct circular profiles consistent with disc-like micelles (Fig. 4B). The high-level background staining over the film regions between these two classes of structure made visualization difficult, especially in the case of smaller structures. By contrast, bicelle preparations embedded in MC films containing PTA provided a pattern of the elongated wormlike structures that now appeared aggregated into “stacks” (Fig. 4C). As with the liposome preparation this observation was consistent with movement of the bicelles across the support after adsorption. Disc-like structures were not clearly shown using PTA/MC.

More distinct images of bicelles were obtained using UA/PTA and UA/STA mixtures in MC (UA/PTA shown in Fig. 4D). In these double-staining methods both wormlike structures and disc-like structures were now well seen, with the borders of these structures taking up electron dense stain. In contrast to the single UA staining, the background staining between the structures was relatively light, enhancing the contrast of the lipid structures. Precipitates were again observed with UA/PTA combinations but could be reduced by lowering the amounts of PTA and/or MC added to the double stain mixture or by substituting PTA for STA (data not shown).

One of the artifacts of negative stain is a patchy display with some of the particles becoming obscured, making sampling difficult and restricting conclusions. We next investigated whether MC films consistently displays bicelle particles across the grid. Over the main body of the support grid we could not find areas where the particles were obscured, although at the extreme periphery where the film meniscus joins the metal loop the films were too thick for clear imaging of the bicelles. Optimum staining was produced using tungsten wire thickness of 0.2 mm.

### 3.3 Nanodiscs

**Fig. 5:**
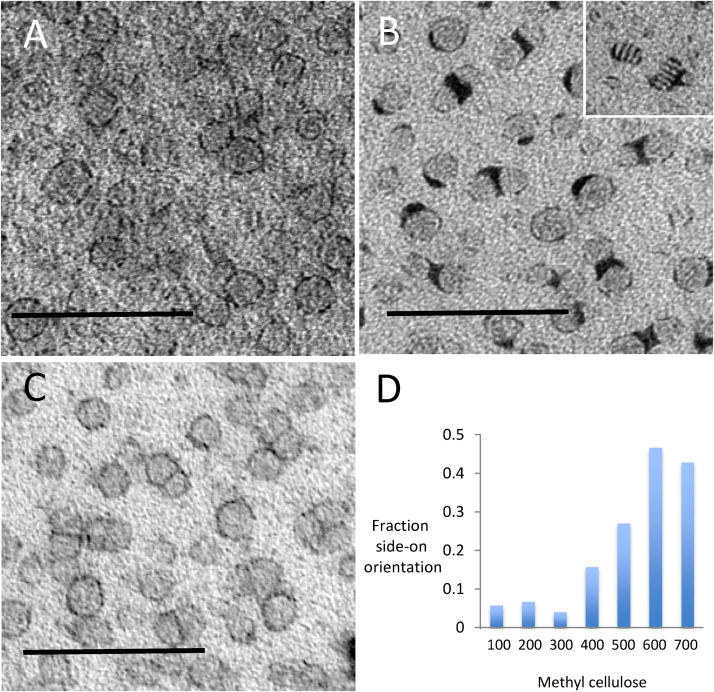
Nanodiscs. Nanodiscs in MC films containing heavy metal stains. UA staining alone (A) provides higher background density compared to PTA (B) and UA/PTA (C). The perimeter of the nanodisc is best displayed in the mixed stain. Clumping occurred when PTA is used alone (B) and is more marked for particles oriented end-on (inset in B). Consistent with support of these structures, increasing the MC content (μl) of the staining mixture increases the fraction of nanodiscs with “end-on” orientation (N = 35, 45, 49, 38, 37, 60 and 70 for each successive category; total, 334) (D). Bars 50 nm.

The nanodiscs used in this study measured approximately 12 nm in diameter (across the disc) and provided an opportunity to test whether UA/PTA and UA/STA stains were able to improve display of smaller lipid-rich structures. UA/MC staining alone produced images consisting of rings of electron density that were inconsistently displayed (Fig. 5A). The intensity of the background stain in the MC was again high compared to that over the body of the nanodisc structures, reducing visualization of nanodisc profiles. Single staining using PTA/MC provided lower background intensity over the MC film with clear staining of the nanodisc periphery (Fig. 5B). Stacking of structures was especially evident when the discs were embedded in the film in end-on orientation, forming “rouleaux”- like assemblies (inset in Fig. 5B). The periphery of the nanodiscs was variably coated with dense accumulations of PTA stain. Again double staining with UA/PTA or UA/STA mix in MC produced the clearest nanodisc morphology (Fig. 5C) producing striking improvement on display of even the smallest nanodisc structures.

**Fig. 6:**
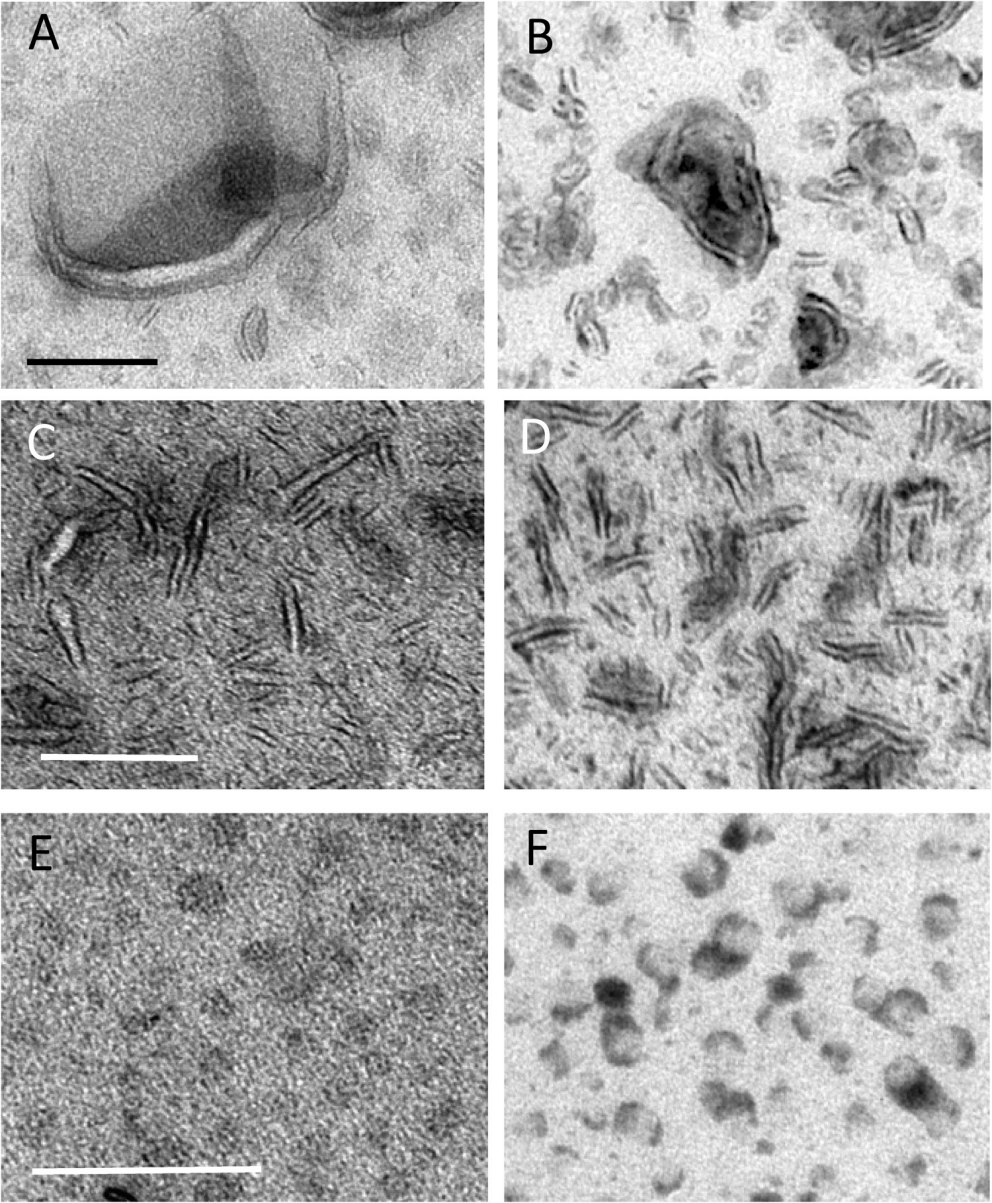
Double staining in sequence. Uranyl acetate and PTA in MC were applied sequentially as described in the methods after absorption of liposomes (A and B), bicelles (C and D) and nanodiscs (E and F). Left hand panels (A, C and E) illustrate results obtained from uranyl acetate followed by PTA staining and right hand panels (B, D and F) results obtained from PTA followed by uranyl acetate. Uranyl acetate followed by PTA produced similar contrast to that obtained using uranyl acetate alone. Using PTA prior to UA produced similar contrast to that obtained with PTA alone, although aggregation of the particles was less evident. Bars for A and B (100 nm); for C and D (100 nm) and for E and F (50 nm).

Stains in MC (UA or PTA) were also used in sequence (Fig. 6). When UA was used first, the clustering artifacts and intense surface staining observed with PTA alone was inhibited, with negative staining of membranes in liposomes became increasingly clear. When PTA was used first, the resulting contrast was either inferior to the double staining method (liposomes and nanodiscs) or similar to the single staining protocol (bicelles). The visualization of structures was however not as clear as with the double staining mixtures, UA/PTA or UA/STA.

### 3.4 Effects of methylcellulose films on lipid rich nanoparticles

Negative stain works by drying down thin films of stain around the particles of interest. Variability in film thickness creates inhomogeneity of display, which sometimes obscures a proportion of the particles. Furthermore, the orientations of particles can be limited due to the thinness of the film. When MC was used, particles were consistently visible across the grid. We also noted that towards the edge of the grid, where the film was thickest, there were more side-on orientations of nanodiscs. Next, we studied the effect of film thickness on nanodisc orientation by varying the concentration of MC in the final staining solution. We found the thickness of the MC films (as indicated by their electron density) could be adjusted and found that the thicker films displayed higher proportions of side-on views (Fig. 5D). In the thinnest films, nanodiscs were almost exclusively seen *en-face*. Interestingly staining with MC/PTA produced stacking of nanodiscs that was observed mostly when the discs were edge on (disc shaped), while in thinner films with *en-face* orientations, this effect was very much less marked (data not shown).

### 3.5 Cryosections

UA/MC embedment was originally introduced to support and facilitate contrast in thawed cryosections (Tokuyasu, 1978, Tokuyasu, 1980, Griffiths et al., 1984). The increased performance of double staining in imaging LRPNs prompted us to examine the effects of double staining on the contrast of ultrathin cryosections. Cryosections of aldehyde fixed RK13 cells and rat liver hepatocytes were contrasted in MC films containing either single UA or double UA/PTA or UA/STA stains. The difference in contrast between single and double stains was not as striking as with adsorbed LRPNs. When compared to UA alone, the double stain showed increases in electron lucency of membrane bound compartments with slight reductions in the cytosolic staining and increases in the density of the mitochondrial matrix. Overall membrane display was therefore enhanced to a minor degree.

## 4 Discussion

Negative stain TEM is a powerful technique for investigating the morphology of macromolecular structures and complexes and is a rapid and accessible method, especially compared to cryo-EM (Harris and De Carlo, 2014). The method is based on the drying of metal salts around the structures of interest forming a “lake” of stain, which provides contrasting and partial support. “Soft” deformable LRNPs such as nanodiscs (Katayama et al., 2010, Imura et al., 2014, Orlando et al., 2014, Akkaladevi et al., 2013), bicelles (Harroun et al., 2005, Vestergaard et al., 2015, van Dam et al., 2004, Rubio et al., 2011b) and liposomes have been imaged with varying success, with their large size compared to the dried film of contrasting agent contributing to drying artifacts. A number of artifacts have been identified previously including low contrast, particle collapse during drying and rouleau formation that has been observed after contrasting with phosphotungstic acid (Zhang et al., 2013).

Amphipathic polymers such as MC bind to a variety of surfaces via multiple short acting interactions and are known to stabilize foams, emulsions, liposomes and solid particles (Nasatto et al., 2015). MC is relatively inert, making it a good candidate agent for stabilizing the LRPNs under study. Importantly, after drying, MC produces films that are thicker than those produced by classical negative stain, and is therefore a useful embedding medium providing structural support. In the pioneering studies of Tokuyasu, such properties were harnessed for preserving the intact morphology of cryosections (approximately 50–100 nm thick; 25–27). This prompted the sporadic use of MC films for embedding and preserving endosomal structures (Lucocq et al., 2001) and exosomes (Raposo and Stoorvogel, 2013). Here we exploited the potential of MC for supporting and preserving lipid rich supramolecular nanoparticles and initially used a standard protocol for MC embedment incorporating uranyl acetate into a hydrophilic film. We found that MC supports a range of lipid structures when single staining with uranyl acetate is used. Importantly the morphology of the structures was well preserved across the whole MC film, right out to the edge where the film starts to thicken, indicating that nearly all the particles were available for examination. This is an important advantage over negative stain and cryo-EM where artifacts can obscure a proportion of the particles.

A related strategy for reducing drying artifacts was to use hydrophilic sugars (Unwin and Henderson, 1975), which were found to be particularly useful for two-dimensional crystals. For example, the inclusion polymers such as trehalose (Chiu et al., 2011), is proposed to stabilize supramolecular lipid structures such as liposomes during drying (Harrigan et al., 1990), and is thought to stabilize lipid head groups by replacing water at the molecule surface via hydrogen bonding. Trehalose may also prevent the gel transition on drying and could act as spacing agent that prevents vesicle fusion.

Heavy metal stains used for negative staining of biological macromolecules can be categorized broadly as anionic or cationic. Uranyl acetate produced a negative staining pattern with high background over the film but also displayed some binding to the surfaces of lipid structures, presumably due to ionic interactions with phosphate moieties of the lipid head groups. In contrast PTA and STA produced intense staining of structure surface, sometimes obscuring details of the structure with only weak background staining. PTA and STA are anionic stains and contain negative charges, and likely bind to positively charged lipids found in the lipid structures (for example DMPC and the PE). Interestingly we found that stacking of structures occurred after the use of PTA and STA stains alone and after adsorption to the EM grid support, suggesting lipid structures can move on the EM grid support. Formation of these so called rouleau is a recognized effect of negative stain protocols, which can be reduced by careful fine tuning of the staining technique (Rames et al., 2014).

Mixing PTA or STA with uranyl acetate produced striking enhancement of lipid structure display, with significant reductions in the staining background compared to uranyl acetate. The mechanisms of staining reactions are likely to be complex, but it appears that uranyl acetate inhibits the interactions of PTA or STA with lipid structures that cause stacking and “rouleau” formation. Uranyl acetate also appears to prevent excessive accumulation of PTA stain at the outer aspect of lipid structures.

The double staining protocol improves on published negative staining of lipid structures. Nanodiscs display very variable contrast in methylamine tungstate negative stain (Katayama et al., 2010), and we find that bicelles and liposomes are now much better contrasted than with PTA or UA alone. In particular, the smallest structures in the bicelle and liposome preparations were now clearly visible, while they were indistinct using either PTA or uranyl acetate alone. The improved visualization is likely to facilitate the quantitative analysis of size distributions. In future it will be interesting to test whether double staining could also improve negative staining techniques on smaller molecular structures such as proteins or nucleic acids, perhaps revealing subtle changes in molecular morphology.

We found that the double staining protocol with PTA produced linear/crystalline precipitates across a range of PTA and uranyl acetate concentrations. These precipitates were not formed at a level that obscured underlying structures substantially, making sampling for quantification possible. These precipitation effects could be reduced by (i) reducing the amounts of PTA, (ii) substituting STA for PTA or (iii) by reducing film thickness. The reduced precipitation seen on substitution of STA for PTA, suggests that phosphorus moieties (possibly interacting with UA) are responsible for formation of the crystalline aggregates.

One potential systematic error of negative stain is the distortion and flattening of structures during the drying procedure. MC produces thicker films and should support larger structures such as LRPNs (Hacker et al., 2016). We tested the degree to which nanodiscs undergo preferential orientation and found more end-on particles when larger amounts of MC were used leading to thicker films after drying. This result suggests that measurement of soft and flexible nanoparticles could be facilitated by embedding particles in thicker MC films (e.g. made with highest amounts of MC and or thicker wire loops).

In summary, we show the MC double staining (UA/PTA or UA/STA) protocol is well suited to the study of a range of supramolecular lipid structures, offering unique advantages over previously reported methods and significant improvements in image contrast. This method should improve routine characterization of lipid nanostructures, which is a crucial part of the quality assurance process. One example is liposomes, for which reproducibility and storability are major issues in producing drug delivery reagents (Sercombe et al., 2015). Through its ease of use, the method should also improve throughput while the comprehensive visualization of particle populations should increase the reliability of quantitative results.
